# Converging or diverging trajectories of mortality under one year of age in the Baltic States: a comparison with the European Union

**DOI:** 10.1186/s13690-021-00598-0

**Published:** 2021-05-13

**Authors:** Agnieszka Genowska, Birute Strukcinskiene, Anita Villerusa, Jerzy Konstantynowicz

**Affiliations:** 1 Department of Public Health, Medical University of Bialystok, Bialystok, Poland; 2 Faculty of Health Sciences, Klaipeda University, Klaipeda, Lithuania; 3 Department of Public Health and Epidemiology, Institute of Public Health, Rīga Stradinš University, Rīga, Latvia; 4 Department of Pediatrics, Rheumatology, Immunology and Metabolic Bone Diseases, Medical University of Bialystok, University Children’s Hospital, Bialystok, Poland

**Keywords:** Stillbirth, Neonatal mortality, Infant mortality, Health inequalities, Macroeconomic factors

## Abstract

**Background:**

Information about trends in perinatal and child health inequalities is scarce, especially in the Eastern Europe. We analyzed how mortality under 1 year of age has been changing in the Baltic States and the European Union (EU) over 25 years, and what associations occurred between changes in macroeconomic factors and mortality.

**Methods:**

Data on fetal, neonatal, infant mortality, and macroeconomic factors were extracted from WHO database. Joinpoint regression analysis was performed to analyze time trajectories of mortality over 1990–2014. We also investigated how the changes in health expenditures and Gross Domestic Product (GDP) contributed to the changes in mortality.

**Results:**

The reduction of fetal, neonatal and infant mortality in the Baltic countries led to convergence with the EU. In Estonia this process was the fastest, and then the rates tended to diverge. The strongest effect in reduction of neonatal mortality was related to the annual increase in health expenditure and GDP which had occurred in the same year, and a decrease in fetal mortality associated with an increase in health expenditure and GDP in the 4th and 5th year, respectively, following the initial change.

**Conclusions:**

These findings outlined convergences and divergences in mortality under 1 year of age in the Baltic States compared with the patterns of the EU. Our data highlighted a need to define health policy directions aimed at the implementation of effective intervention modalities addressing reduction of risks in prenatal and early life.

## Background

Mortality rates under 1 year of age, as the most severe adverse birth outcomes, are important indicators of population health and the distribution of human welfare. They are also regarded as useful measures of the quality of health care for mother and child [1, 2]. In developed countries, a significant decrease has been reported in the indices of fetal, neonatal and infant mortality during recent decades. This trend was associated with not only an improved prenatal care and advanced perinatal care, but also with a large-scale implementation of oxygen therapy, and predominant use of corticosteroids and antibiotics [3, 4]. Though the situation has been considerably improved, the mortality under 1 year of age still confers an important public health challenge.

In the European Union (EU), mortality under 1 year of age is the lowest in the world [5, 6]. However, inequalities in perinatal and child health between individual member countries continue to be reported. In general, the highest mortality rates were approximately 4-fold higher than the lowest ones. Given that in 2014 fetal mortality rates ranged from 2.7 to 10.2 per 1000 total births, whereas infant mortality rates ranged from 2.0 to 8.1 per 1000 live births [7]. Cross-comparisons between countries within the EU may be difficult at the methodological level as there are essential individual differences in registration criteria used for live births and stillbirths. Nevertheless, the proportions of mortality remain even when these differences are taken into account [8–10]. The adverse birth outcomes were reported particularly in Central and Eastern European populations in comparison to Western countries. The inequalities in perinatal and child health require a special focus in the Baltic States (Estonia, Latvia, and Lithuania) which demonstrate a unique background and offer special conditions for evaluation of health outcomes.

The three Baltic States underwent a systemic change and became reorganized in the early 90’s of the twentieth century when the states gained independence after the fall of the Communist regime in the Soviet Union. Although the transformation process from socialism into the market economy was similar in the region and took place within a short time-period, the socio-economic conditions had been variable and differed across individual countries [11, 12]. The denationalization and liberalizing of the economy in Estonia progressed much faster than in other Baltic countries, and the process has resulted in increasing inequalities between countries in the region. In Latvia the precipitate changes regarding reconstructing of the industry and increasing unemployment occurred in the mid 90’s. Contrastingly, Lithuania was proceeding with the stepwise economic changes gradually within a longer time [12, 13]. Along with differing living conditions, there were significant differences in health care provisions and standards. The changes and fluctuations also had an impact on women’s health and reproductive health and subsequently they affected fetal, neonatal and infantile health status [14].

In order to assess the trajectories of perinatal and child health in the Baltic states within the last 25 years, the data derived from World Health Organization (WHO) and core indicators provided by Euro-Peristat were used [15]. Noticeably, in the early 90’s the rates of mortality under 1 year of age were high in all Baltic countries and thereby the decrease of the indices was faster. We hypothesized that the level of mortality under 1 year of age in the Baltic states may become similar to the average level typically found in the developed EU countries. The European Union may serve as an appropriate comparator because the large numbers of live births reaching about 5 million per year ensure a good reliability of the analyses [5, 7]. In this study, we analyzed how mortality under 1 year of age has been changing in the Baltic States and the European Union over 25 years, and what associations occurred between changes in macroeconomic factors and mortality.

## Material and methods

This study was performed according to the mortality under 1 year of age in Estonia, Latvia, Lithuania and EU members for the period 1990-2014. We analyzed the rates of mortality for the most recent data, which was available for the Baltic countries and the EU in the same years (from 1990 or 1991 to 2014). This data is based on perinatal death certification and medical death certification and updated annually from national civil registration systems. The quality data of mortality under 1 year of age has been evaluated by the WHO [16].

The general concept for the analysis were indicators approved and used by the Euro-Peristat [15]; this being an essential part of the EU’s Health Monitoring Programme and now has representation from 31 countries across Europe and a network of experts. Core indicators estimating perinatal and child health were included in the analysis along with patterns of mortality under 1 year of age. For example:

- (C1) Fetal mortality rate is the number of fetal deaths at or after 22 completed weeks of gestation in a given year per 1000 live and stillbirths in the same year.

For comparisons in fetal mortality rates between Baltic countries and the EU, the estimated mean fetal mortality rate was derived from 16 European states in which the registration criteria for fetal death are lower or equal to 22 weeks of gestation age. The inclusion of 16 EU countries (i.e. Austria, Czech Republic, Denmark, Estonia, Finland, Germany, Hungary, Ireland, Italy, Latvia, Lithuania, Netherlands, Poland, Portugal, Slovenia, and United Kingdom) led to a more reliable and accurate estimation of fetal mortality rates [17].

- (C2) Neonatal mortality rate - the number of neonatal deaths (day 0 through 27) after live birth in a given year per 1000 live births in the same year.
- (C3) Infant mortality rate - the number of deaths (day 0-364) after live birth in a given year per 1000 live births in the same year.

The rates C2 and C3 were presented for all births at or after 22 completed weeks of gestation.

To evaluate the effect of the macroeconomic situation in EU countries on mortality, we included data on total health expenditures and Gross Domestic Product (GDP) measured per capita. We defined total health expenditures as current spending by both public and private sources on medical services and goods, public health and prevention programmes, and administration, expressed in Purchasing Power Parity (PPP) in US dollars [6]. GDP represents the sum of gross value added by all resident producers in the economy plus any product taxes and minus any subsidies not included in the value of the product, expressed in current international United States dollars (US$) [7]. We have studied the relationship between mortality under 1 year of age, and macroeconomic factors since 1995 due to a lack of data on health expenditures over 1990-1994. Comparable data for indicators of perinatal and child health, and macroeconomic situation were obtained from WHO European Health Information Gateway – Health for All explorer [7].

### Statistical analysis

To examine the changes associated with mortality under 1 year of age during the last 25 years, the Joinpoint regression analysis was performed allowing for significant changes in trends during the period to be detected. The applied analytic method automatically identified number and locations of Joinpoints during the observation period, restricting their number to 3, resulting in up to 4 linear segments in trend line. Estimated linear segments are presented as annual percentage change (APC). A summary measured over years 1990-2014 showed the annual average percent change (AAPC) with a 95% confidence interval was calculated as a weighted average of partial trends APCs [18].

To analyze relationships in the time period 1995-2014 between changes in macroeconomic situation and fetal, neonatal and infant mortality, we took into consideration assumed time-lag between cause and result and we used the following linear fixed effects statistical model:

$$ \Delta  {Y}_{c,t}-\overline{\Delta  {Y}_c}=\alpha +\beta \left(\Delta  {X}_{c,t-l}-\overline{\Delta  {X}_c}\right)+{\gamma}_ct+\kappa {K}_{c,t}+\lambda {L}_{c,t}+{\varepsilon}_{c,t} $$

c – country

t – time (year)

l – time lag (years)

Y – dependent variable (fetal, neonatal and infant mortality)

X – main independent variable (health expenditure and GDP)

K, L – demographic structure variables (percentage of people below 15 and above 65 years of age)

The model included annual changes in indicators of mortality (standardized between countries by subtracting country average) as a dependent variable. Main independent variables of interest were annual changes in macroeconomic factors (standardized between countries by subtracting country average). Regression coefficient for those variables (β) describes relationships between the economic factor change and health outcome response. We implemented time-lag from 0 to 5 years for macroeconomic variables to account for assumed delay between economic impulse and health outcome. Each model also included country-specific rate of acceleration (γ_c_). The model was also adjusted for country demographic structure by including percentages of population below 15 years of age and above 65 years of age. Robust standard errors were used to account for correlation of data within countries. Similar statistical models based on annual changes rather than actual value of variable have been used in other health-economic studies [19, 20].

Statistical analysis was performed using Joinpoint Regression Program 4.7.0.0-2019 and IBM® SPSS® Statistics for Windows, Version 20.0 (IBM Corp., Armonk, NY, USA). Statistical significance (I-type error probability) was set at α = 0.05.

## Results

**Fig. 1:**
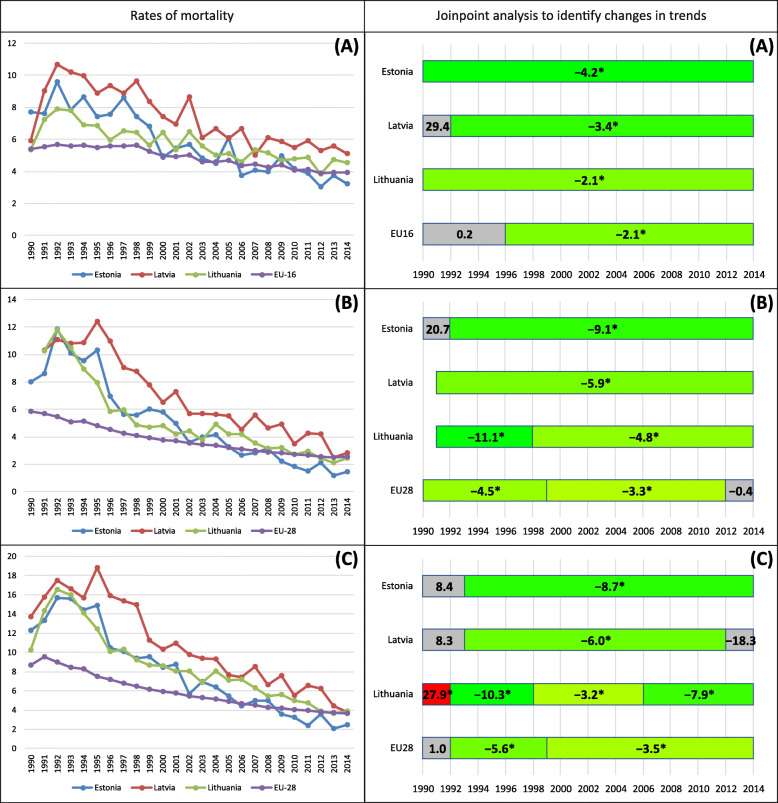
Mortality under one year of age in the Baltic States and the European Union, 1990-2014. (**A**) Fetal mortality. (**B**) Neonatal mortality (data were not available in year 1990 for Latvia and Lithuania). (**C**) Infant mortality EU-16: Austria, Czech Republic, Denmark, Estonia, Finland, Germany, Hungary, Ireland, Italy, Latvia, Lithuania, Netherlands, Poland, Portugal, Slovenia, United Kingdom *Statistically significant trend at *p* < 0.05

In the Baltic states, there were disparities in rates of fetal, neonatal and infant mortality, with the highest levels in Latvia in comparison to Estonia and Lithuania. As shown by analysis of the indicators of mortality under 1 year of age and the average annual percent decline in these rates, the gaps in mortality between the Baltic states and the EU narrowed during the years 1990-1992 and 2012-2014 (Table 1, Fig. 1). In the years 1990-2014, the largest reduction of mortality rates in the Baltic states occurred in Estonia regarding all analyzed mortality rates: neonatal (by 8.4% per year), infant (by 7.9% per year) and fetal (by 4.2% per year). Decrease of mortality in Estonia led to convergence of coefficients with the EU level. For the first time fetal mortality rate approached in 2000, and neonatal and infant mortality in 2002. At the end of the analyzed period in 2014, a divergence of mortality patterns was found in Estonia, i.e., all indicators of mortality were below the EU level. In Lithuania and Latvia, the change of mortality rate was slower compared to Estonia, while in Lithuania neonatal mortality rate exceeded the average of the EU in 2010, and infant mortality rate in 2013. In Latvia, the rates of neonatal and infant mortality were similar to the EU only at the end of the observation period in 2013-2014. In the case of fetal mortality, Lithuania and Latvia in 2014 achieved slightly higher ratios than the EU level (per 1000 total births: by 1.17 and by 0.61, respectively). In the Baltic countries and the EU, reduction in the rate of fetal mortality was slower compared to the neonatal and infant mortality.

**Table 1:** Changes in mortality under one year in the Baltic States and the European Union, 1990–2014

Indicator		Rates		Trend 1990–2014	Gap between Baltic States and European Union	
		3-years average (1990-1992)	3-years average (2012-2014)	AAPC (95% CI)	3-years average (1990-1992)	3-years average (2012-2014)
Fetal mortality rate [n/1000 total births]	Estonia	8.31	3.35	−4.2* (− 4.9, − 3.4)	2.77	− 0.58
	Latvia	8.53	5.34	−2.6* (− 3.5, −1.8)	2.99	1.41
	Lithuania	6.82	4.38	−2.1* (−2.7, −1.5)	1.28	0.45
	EU-16	5.54	3.93	−1.7* (− 1.9, − 1.5)		
Neonatal mortality rate [n/1000 live births]	Estonia	9.50	1.60	−8.4* (−9.4, −7.5)	3.83	−0.94
	Latvia	10.70 (1991-1992)	3.18	−5.9* (−6.7, −5.2)	5.03	0.64
	Lithuania	11.05 (1991-1992)	2.35	−6.3* (−7.1, −5.5)	5.38	− 0.19
	EU-28	5.67	2.54	−3.6* (−3.8, −3.4)		
Infant mortality rate [n/1000 live births]	Estonia	13.37	2.69	−7.9* (−8.8, −7.0)	3.88	−1.05
	Latvia	15.66	4.84	−5.6* (−6.4, −4.8)	6.17	1.10
	Lithuania	13.70	3.81	−5.5* (−6.2, −4.8)	4.21	0.07
	EU-28	9.49	3.74	−4.0* (− 4.2, −3.8)		

EU-16: Austria, Czech Republic, Denmark, Estonia, Finland, Germany, Hungary, Ireland, Italy, Latvia, Lithuania, Netherlands, Poland, Portugal, Slovenia, and United Kingdom

*Statistically significant trend at *p* < 0.05

Results of the analysis of partial trends indicated the dynamics of change in the Baltic states and the EU, especially in neonatal and infant mortality (Fig. 1). In the initial period from 1990 until the turn of 1992/1993, insignificant mortality trends were observed in Estonia (neonatal, infant), Latvia (fetal, infant), while in Lithuania the trend of infant mortality by 27.9% per year increased significantly. After 1993, there were decreasing mortality trends in all Baltic countries, but in Lithuania until 1998, neonatal and infant mortality was decreasing at the fastest rate (by 11.1% per year and by 10.3% per year, respectively). In Latvia, after 2012, the rates of infant mortality stagnated.

Compared to the EU, the Baltic states had relatively low health expenditure and GDP per capita, given the best macroeconomic situation occurred in Western Europe, i.e. Austria, Germany, Luxembourg (Table 2). In the observed period 1995–2014, health expenditure and GDP increased in all EU countries, but its pace differed between EU countries. The largest increase in health expenditure and was recorded in Romania and Bulgaria, slower growth was observed in Greece and Italy. In the Baltic states GDP increased by as much as 7-fold, the smallest increase occurred in Germany and United Kingdom.

**Table 2:** Macroeconomic factors in the European Union countries in year 1995 and year 2014

Calendar year	Health expenditure [PPP in US$ per capita]		Gross Domestic Product [current international US$ per capita]	
	1995	2014	1995	2014
Austria	2250	5039	30,253	51,323
Belgium	1714	4392	28,566	47,439
Bulgaria	290	1399	1554	7853
Croatia	549	1652	4795	13,467
Cyprus	731	2062	15,098	27,341
Czech Republic	898	2146	5765	19,745
Denmark	1875	4782	35,351	62,426
Estonia	397	1668	3044	19,941
Finland	1480	3701	26,273	49,915
France	2102	4508	27,038	42,955
Germany	2280	5182	31,730	47,903
Greece	1267	2098	12,959	21,674
Hungary	659	1827	4481	14,118
Ireland	1193	3801	19,177	55,503
Italy	1559	3239	20,596	35,397
Latvia	221	940	2329	15,725
Lithuania	332	1718	2169	16,555
Luxembourg	2189	6812	52,831	119,173
Malta	899	3072	9114	26,181
Netherlands	1800	5202	28,885	52,157
Poland	407	1570	3683	14,342
Portugal	1017	2690	11,783	22,078
Romania	184	1079	1660	10,020
Slovakia	505	2179	4799	18,595
Slovenia	972	2698	10,691	24,021
Spain	1193	2966	15,430	29,600
Sweden	1745	5219	29,914	59,180
United Kingdom	1350	3377	22,756	46,412
EU-28	1442	3519	19,833	36,552

*PPP* Purchasing Power Parity, *US$* United States dollars

As shown in Table 3, macroeconomic factors were associated with mortality under 1 year of age. The relationships between the change in health expenditure and the change in fetal mortality persisted over 5 years, but the strongest reduction in fetal mortality associated with the annual increase in health expenditure occurred with an interval of 4 years following the initial change (*β* = − 0.1544 per 100 US$ increase). Reduction in fetal mortality also occurred with an annual increase in GDP after a 5-year delay (*β* = − 0.0026 per 100 US$ increase). Lag analysis of health expenditure showed that these associations with neonatal mortality were significant and maintained for 4 years, and the relationship with GDP was in year 0 to 1, and 4 to 5 years. The strongest effect of reducing neonatal mortality was related to the annual increase in health expenditure which occurred in the same year (*β* = − 0.1641 per 100 US$ increase), a similar relationship in the same year was also found with GDP (*β* = − 0.0049 per 100 US$ increase). The change in the size of the health expenditure effect was associated with infant mortality in the same year (*β* = − 0.0788 per 100 US$ increase) and changes in GDP did not occur in significant relationships with infant mortality.

**Table 3:** Associations between macroeconomic factors increase with mortality change in the same year and considering 1 to 5 year lag-time

Time-lag in years	Expected impact of 100 US$ per capita annual rise in health expenditure on mortality (*β* with 95 % CI)	Expected impact of 100 US$ per capita annual rise in GDP on mortality (*β w*ith 95 % CI)
Fetal mortality		
0	−0.0972 (−0.1698, −0.0246)**	−0.0023 (−0.0052, 0.0006)
1	−0.1139 (−0.1825, −0.04531)***	−0.0015 (−0.0044, 0.0001)
2	− 0.1241 (− 0.1932, − 0.0549)***	− 0.0020 (− 0.0046, 0.0006)
3	− 0.1408 (− 0.2149, − 0.0666)***	−0.0020 (− 0.0051, 0.0012)
4	− 0.1544 (− 0.2072, − 0.0101)***	−0.0027 (− 0.0057, 0.0003)
5	− 0.1338 (− 0.1865, − 0.0811)***	−0.0026 (− 0.0048, − 0.0004)*
Neonatal mortality		
0	− 0.1641 (− 0.2298, − 0.0985)***	−0.0049 (− 0.0068, − 0.0030)***
1	−0.1325 (− 0.2016, − 0.0633)***	−0.0045 (− 0.0064, − 0.0025)***
2	−0.0567 (− 0.1036, − 0.0099)*	−0.0003 (− 0.0019, 0.0014)
3	− 0.1156 (− 0.1860, − 0.0452)***	−0.0018 (− 0.0040, 0.0004)
4	− 0.1110 (− 0.1801, − 0.0419)**	−0.0027 (− 0.0043, − 0.0010)***
5	−0.0734 (− 0.1496, 0.0028)	−0.0026 (− 0.0044, − 0.0001)**
Infant mortality		
0	− 0.0788 (− 0.1212, − 0.0364)***	−0.00003 (− 0.0014, 0.0013)
1	0.0330 (− 0.0072, 0.073)	−0.0021 (− 0.0055, 0.0013)
2	0.0102 (− 0.0384, 0.0589)	−0.0001 (− 0.0014, 0.0013)
3	− 0.0100 (− 0.0540, 0.0339)	0.0010 (− 0.0012, 0.0031)
4	−0.0269 (− 0.0779, 0.0242)	−0.0002 (− 0.0020, 0.0017)
5	0.0174 (− 0.0376, 0.0723)	0.0001 (− 0.0011, 0.0012)

β – the impact of annual changes in health expenditure and GDP on mortality controlling for population structure (proportion of population below 15 years of age and proportion of population above 65 years of age) and controlling for inter-country differences in health expenditure and GDP

***p ≤ 0.001; **p ≤ 0.01; *p ≤ 0.05

## Discussion

Mortality under 1 year of age significantly decreased in all countries, and a convergence of the rates continued to be observed between the Baltic states and EU-28. This convergence occurred most rapidly in Estonia, where the rates of fetal mortality exceeded the data for EU-16 in 2000 for the first time ever, and the neonatal and infant mortality − in 2002. In subsequent years, the trend to divergent values was observed. In Lithuania, the convergence of mortality patterns occurred slightly later, the neonatal mortality rate exceeded the average of EU in 2010, while that of infant mortality did so in 2013. In Latvia, the mortality rate approached the average EU level at the end of the analyzed period, but did not yet exceed it. Time-lag analyses suggested that in the EU countries the increase in health expenditure and GDP was linked to a reduction fetal mortality with the strongest effects after a 4 and 5-year delay. Growth of health expenditure and GDP diminished neonatal mortality, especially in the same year.

The evidence changes related to decrease in mortality under 1 year of age in the Baltic states was reported by Rajaratnam et al., showing a decline in rates of neonatal and infant mortality in a cross-comparison across 21 regions of the world [5]. There have also been published studies reporting inconsistent results or suggesting opposite trends in fetal mortality, being either decreasing or increasing [3, 15, 21]. In all the Baltic states, following the political and economic transformation, the Program of Perinatology was successfully started with the essential financial support of the Swiss government. Thereby, implemented organizational and structural changes in mother and neonatal care, including intensive care units and medical transport system, have significantly improved birth outcomes [14, 22]. There was a possibility that the preponderance regarding better birth outcomes in the Baltic states compared with the rest of EU resulted, at least partly, from a close cooperation with Scandinavian experts in the field and shared principles of good clinical practice in the perinatal and obstetric care [14]. Nordic countries have prominent results on maternal and child health indicators, and low neonatal mortality rates [5, 8, 23, 24]. Noticeably, in Estonia the mortality rates under 1 year of age decreased most rapidly suggesting some country-specific factors. One of the possible explanations was the alleviation of income inequality and some beneficial effects of the maternal educational level [11, 25]. It is worth emphasizing that Estonia also significantly improved the health status of adult women as a result of health care activities and reduction of harmful lifestyle factors [26], which could have contributed to the improvement of birth outcomes. This dynamic evolution in Estonia after 2006 led to the divergent rates of mortality under 1 year of age with the EU, and Estonia achieving the level similar to the high-income countries. The divergence noted in Estonia provides some assumption that such a continuous improvement is also possible in Lithuania and Latvia.

The general health status among the population was worsening in the early 90’s. After 1994, the situation turned into an improvement, but there was still a significant gap between the Baltic states and the EU in health status. In the Baltic countries mortality due to cardiovascular diseases, cancer, and suicides reached the highest rates in comparison with other EU countries [27, 28]. Significant improvements in perinatal and children health in the Baltic region give hope for faster improvement as well in the health of the whole population.

Our results showed that there is a concern about a slower velocity of the fetal mortality reduction compared to the neonatal and infant mortality in the Baltic countries and the EU. Around 80% cases of fetal deaths are antepartum stillbirths and that could be associated with a delay in the first antepartum visit or suboptimal care, and coincident poor socioeconomic status and harmful factors such as smoking [29, 30]. Our results showed that in the EU countries, capability to further the reduction of fetal and neonatal mortality through healthcare activities are still present. Moreover, an increase in GDP can bring benefits with improved fetal and neonatal health. A possible explanation of the results is that GDP growth contributes to an overall increase of socioeconomic resources, and to the increase of socio-economic status. With the larger GDP growth, countries are more likely to implement health care programs that improve fetal and neonatal health. These factors may be modifiable to a great measure, basically through the targeted prevention programs in the health care system and high quality hospital care services [26, 30–32]. In addition, the parallel investments in social policies and education for young women and families at high risk are important [4, 33–35]. It is important to mention that the fetal and neonatal health is an investment to the general health of the entire society and therefore may considerably influence and shape the health status of future generations.

### Strengths and limitations

The strong side of this study was a focus on core indicators from Euro-Peristat used for the monitoring of trajectories mortality under 1 year of age in the Baltic states and the EU in the 25-year period (from 1990 to 2014). This study showed the areas of progress in fetal, neonatal and infant health in all countries, and also from year 2012, stagnation in the rate of infant mortality in Latvia and neonatal mortality in the EU. In addition, the strong side of the paper is comparability of mortality rate in Estonia, Latvia and Lithuania, where fetal mortality rates are lower or equal at 22 weeks of gestational age. However, in the investigated countries the recorded stillbirths exclude abortions of pregnancy which are recorded in another system [8, 9]. The above uniform criteria allows addressing the issue whether the decreasing trend of fetal mortality resulted from the decrease of stillbirth cases or because of the decreasing trend of terminating pregnancies. The study revealed that while excluding from our analysis 12 countries of the EU and using the data recording the criterion of over 22 weeks of gestation, the trend of fetal mortality rates decreased in EU-16 and those trends were similar to the Baltic states. In the EU-28, the trend remained stable [7] which caused us to conclude that exclusion of 12 countries gave the possibility for more exact estimation of similarities in fetal mortality trends, and converging or diverging trajectories between the Baltic countries and the EU.

This study showed important implications for policy; that improving the macroeconomic situation appears to diminish mortality under 1 year of age in the EU. It is important in terms of low fertility levels and may be a guide in the decision-making process regarding the distribution of resources aimed at intervention for child health [36]. There is a need for government action to increase well-being and access to appropriate obstetric care. These factors can contribute not only for improvement in child health, but also for long-term effects throughout the lifecycle related to better adult health and increased productivity in the labor market.

A limitation of the study is the issue of data quality over time. The possibility of mismatches is still possible; even if mortality data and statistical information in the Baltic countries is used by the global health organizations and databases (for instance, the WHO) and are generally considered reliable and internationally comparable [37]. Some limitations still could be linked with differences in definitional arrangements and registration as well as hospital policies regarding delivery and neonatal unit admission; particularly around the limits of viability in EU countries [24, 38]. Other issue is the interpretation of lag-time analysis, based on mortality and macroeconomic factors, in which aggregated data level may have limited conclusions on potential causal relationship at an individual level. Independent variables used in the ecological study represent mortality and macroeconomic factors observed on the population level, and not the characteristics of individuals [39]. An increase in health expenditure and GDP does not always translate into improvement of health outcomes, although the economic progress and stimulation was independently associated with mortality reduction. Furthermore, caution is advised while interpreting lag-time analysis because these results may be underestimated due to lacking or incomplete data on mortality, especially in newborns. Nevertheless, our study incorporated mortality reports from all EU countries based on high quality standard and reliable procedures of data collection.

In our analysis we focused on macroeconomic factors with regard to controlling factors, however, the number of variables potentially influencing was restricted. For instance, medical services in gynaecological and obstetric care or prevention programs have not been examined here because of lack of adequate data. Although health expenditure plays an important role in shaping the health of the population, their distribution into individual categories varies between countries [6]. However, the effectiveness of health care, particularly in the context of improvement of perinatal and child health, appears as important as health expenditure per se. Noticeably, a relatively unfavourable health situation may also affect countries with high health expenditure, e.g. US. Thus, high expenditure may not necessarily be always projected into enhancement of public health status [5, 6]. On the other hand, it has been shown that the Baltic states achieved strikingly good health outcomes despite relatively low expenditures. These associations prove that some other (or unknown) factors connected with intervention programs and quality of health care services may play a role in moderating the effectiveness of the healthcare expenditure. Our results could be explained, at least partly, by confounding effect of the differences in clinical practice and setting, regarding the venue of delivery (i.e., public or private hospital, and outside of the hospital). There is evidence that the venue of delivery is associated with mortality under 1 year of age [40–43]. Adverse birth outcomes were prevalent rather in the public hospitals compared with the private services. Noticeably, the discrepancies resulted from suboptimal allocation of women to antenatal care in public hospitals in terms of perinatal mortality risk and/or delayed medical care [40]. Furthermore, it has been reported that unplanned out-of-hospital birth may be associated with an increases perinatal mortality, due to limited accessibility to certain urgent medical interventions or inadequate antenatal care [41–43]. Finally, the duration of the mother’s stay in the hospital may have been associated with birth outcomes [44], but the data were not available in the analyses, and might be included in future research agenda.

## Conclusions

Within the last 25 years, the Baltic countries experienced several beneficial changes regarding fetal, neonatal and infant health, and this evolution has led to a convergence with the patterns of the European Union. Furthermore, the process proceeded most rapidly in Estonia and ultimately was reversed into the divergent trend. The inequalities in perinatal and child health existing between European Union countries indicates a need of implementation of effective intervention modalities aimed to reduce risks in prenatal and early life. These activities may be an investment in human and social capital, and may help reduce health inequalities resulting from the child health improvement.

## Data Availability

The datasets generated and/or analyzed during the current study are publicly available.

## Abbreviations

APC: Annual percentage change
AAPC: Average percent change
EU: European Union
GDP: Gross Domestic Product
PPP: Purchasing Power Parity
US$: United States dollars
WHO: World Health Organization
